# Supplementary material for: Nitrate Utilization Promotes Systemic Infection of Salmonella Typhimurium in Mice
Source: Int J Mol Sci. 2022 Jun 29;23(13):7220. doi: 10.3390/ijms23137220 (PMC9266322; doi:10.3390/ijms23137220)
Supplement: Supplementary file 1 [file ijms-23-07220-s001.zip › ijms-1792597-supplementary.pdf]

Supplementary Material

Nitrate Utilization Promotes Systemic Infection of *Salmonella* Typhimurium in Mice

Wanwu Li [1,2], Linxing Li [1,2], Xiaolin Yan [1,2], Pan Wu [1,2], Tianli Zhang [1,2], Yu Fan [1,2], Shuai Ma [1,2], Xinyue Wang [1,2], and Lingyan Jiang *

[1] The Key Laboratory of Molecular Microbiology and Technology, Ministry of Education, Nankai University, Tianjin 300457, China; wanwuli@mail.nankai.edu.cn (W.L.); lilinxing19@mail.nankai.edu.cn (L.L.); 2120211278@mail.nankai.edu.cn (X.Y.); wupan2016@mail.nankai.edu.cn (P.W.); 1120180080@mail.nankai.edu.cn (T.Z.); 1120170077@mail.nankai.edu.cn (Y.F.); 2120191113@mail.nankai.edu.cn (S.M.); wangxinyue1120200566@mail.nankai.edu.cn (X.W.);

[2] Tianjin Key Laboratory of Microbial Functional Genomics, TEDA Institute of Biological Sciences and Biotechnology, Nankai University, Tianjin 300457, China

* Correspondence: jianglingyan@nankai.edu.cn

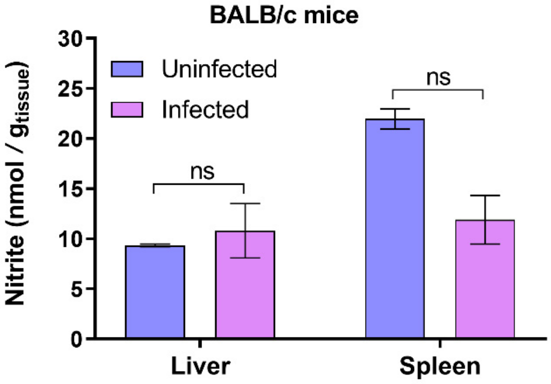

Figure S1. Nitrite levels are not significantly altered during *S.* Typhimurium systemic infection. Nitrite levels were tested in the livers and spleens of BALB/c mice that were infected or mock-infected with *S.* Typhimurium WT strain after 5 days post-infection. Data were generated from three independent experiments and are presented as the mean ± SD. *p*-values were determined using unpaired Student's *t*-test (ns, not significant).

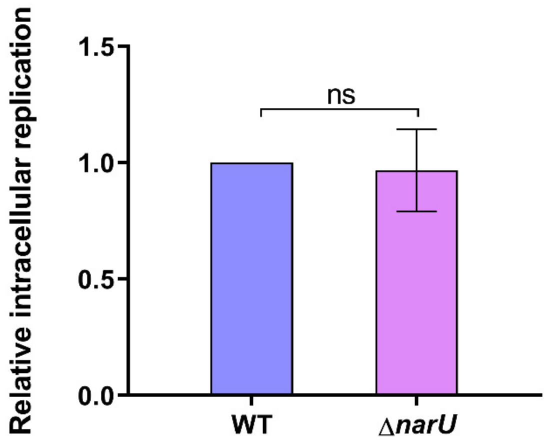

Figure S2. The mutation of *narU* did not significantly influence the replication ability of *S.* Typhimurium in macrophages. Replication of *S.* Typhimurium WT and Δ*narU* in RAW264.7 cells. The bacterial replication ability was determined according to the ratio of the number of intracellular bacteria at 20 h post-infection to the number of bacteria at 2 h post-infection. Data were generated from three independent experiments and are presented as mean ± SD. *p*-value was determined using unpaired Student's *t*-test (ns, not significant).

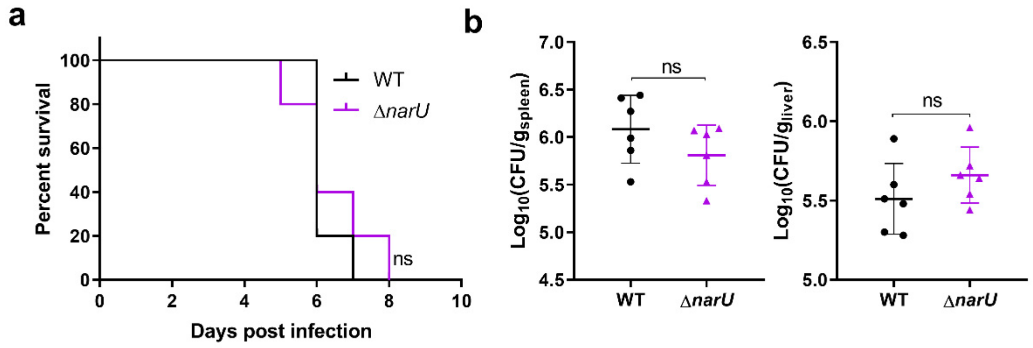

Figure S3. The mutation of *narU* did not significantly influence the systemic virulence of *S.* Typhimurium. (a) Survival curves for mice infected i.p. with the *S.* Typhimurium WT or Δ*narU*, n = 5 mice/group. (b) Liver and spleen bacterial burdens in mice infected with the *S.* Typhimurium WT or Δ*narU* at day 5 post-infection. n = 6 mice/group. (a and b) Data were combined from two independent experiments and are presented as mean ± SD. *p* values were determined using log-rank Mantel–Cox test (a) or Mann–Whitney U test (b) (ns, not significant).

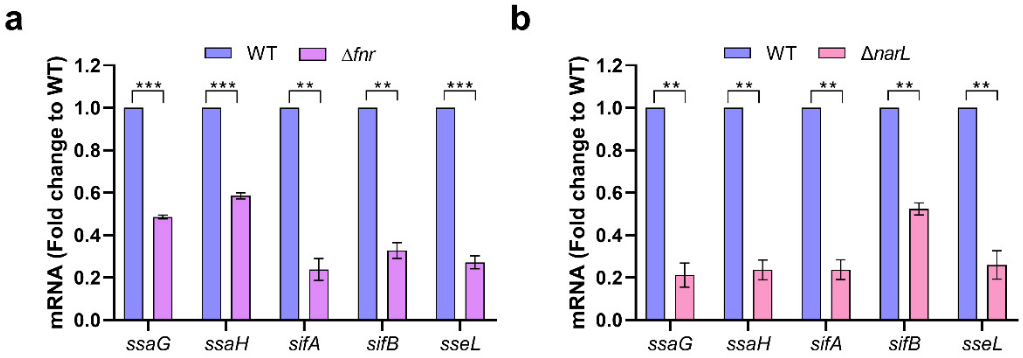

Figure S4. Mutation of *fnr* or *narL* decreased the transcription of T3SS2-related genes. (a and b) qRT-qPCR analysis of the mRNA levels of 5 T3SS2-related genes in S. Typhimurium WT, Δ*fnr* mutant (a), or Δ*narL* mutant (b). Bacteria were grown in N-minimal medium for 6 h prior to collection. Data were generated from three independent experiments and are presented as mean ± SD. *p*-values were determined using unpaired Student's *t*-test ($^{**}p < 0.01$; $^{***}p < 0.001$).

Table S1. Primers used in this study

Targets	Primer sequences (5'-3')
Primers for qRT-PCR analysis	
16S rRNA	ACTGGCAGGCTTGAGTCTTGTAGA
	GGCACAACCTCCAAGTAGACATCG
sifA	AGGACATTTAGATGGGTGGAAAGCG
	TATGTGGGTATGCGGTGGTGGTAT
sifB	GTTGCTTGTTCCCTGAGCGGTTA
	GTCAATAGCTGTTACACCTGCCTGG
ssaG	ATATGCTCTCCCACATGGCGCACCA
	GCGCTTTAATCATCGATTCTGGGTTGAGCA
ssaH	CGGGCGTTAACCATAGCCTGATTT
	TGGTGCAGGAAATAACAGACGCAG
sseL	ACAGGAGATCACTGGCTTCTCTGTT
	CCACAGCCGTTGGGTACATTGTT
narK	ATCCTCATCGTGCCCTGCGTAT
	TATTGCCCAGACCACCGTTCAGA
narG	CGTTCTAACCTGCTTGGGTCTTCC
	CATTGTCACGCCACTCCACTTCTT
napF	CATCGTTGCCAGGACAGTTGTGA
	GCATGGTGGTTCTCCGCTTTGAT
Primers for the establishment of mutant strains	
Δ*narK*	ATATCAACTTACCTTCGGCAGTAAACCCTAATGTGGCAGACATCAAATC
	AAGAATCAGAGGTGTCTGTGTAGGCTGGAGCTGCTTC

	GTATGTTGTAACTAAATACAGATAAAAAAAAGCGCGGTCTAACGCCGC
	GCAAAGGATAATCAAAAGCATATGAATATCCTCCTTAG
Δ*hmpA*	TGCAAGGGTATTTTTATAAGATGCATTTGATATACATCATTAGATTTTCA
	CATAAAGGAAGCACGTGTGTAGGCTGGAGCTGCTTC
	GAACGGGGAGGAAAACGGGCGTTCGCCTTAACGATAACGCCCGTTTTT
	TCAGAGGATTTGTTGCAACATATGAATATCCTCCTTAG
Δ*narU*	GAGCGCGTGACGCTCTGTCTCTTTTTTGTGTTTTTCTTCAAATGATATGCG
	CATGTGAGGGGTAAAGTGTAGGCTGGAGCTGCTTC
	TTCCGCCATCCTGTCGATTTGGCGTTAAACCATTACCGGATGGCCGTCCT
	ACAGGTGCGTATGTTGCATATGAATATCCTCCTTAG
Δ*fnr*	AGACTTACGCGCTCACCAAAAAGATGTTAAAATTGACAAATATCAATT
	ACGGCTTGAGCAGACCTGTGTAGGCTGGAGCTGCTTC
	CCAGATCAATAAATGAGAAAAATTTAACGATATGGCAGAAGATAACAT
	CAATGGTTTAGCTGACGCATATGAATATCCTCCTTAG
Δ*narX*	GCCGATTGACGCCCCTCTTTTTTGCTACGTTTTTTCGGCGACATTACCCC
	GAAGAAAGAAGGTAACGTGTAGGCTGGAGCTGCTTC
	GCTGCTTTACACCCGTGCGTAGCATCGGATGGTCATCGATTAACAGGAT
	GGTTGCCGGTTCCTGACATATGAATATCCTCCTTAG
Δ*narL*	CGGAACAGAGGTCACTGTTACTTTTATTCCAGAAACAAACTTCACAGA
	AACCCAGGGAGATACCCGTGTAGGCTGGAGCTGCTTC
	TGACTGAACCGTTTATCACGATGCCGGACGATAATCCGCATTGGCAACC
	GTTCCAGGAGCAATAACATATGAATATCCTCCTTAG
Primers for the pET-28a-*narL* plasmid construction	
	CCATGGGCATGTTTAAACGGTGTTTCTCTCCTCTTACG
	CTCGAGAAAGATGCGTTCCTGATGTACCCAG
Primers for the amplification of DNA sequences used in EMSA experiments	
narK	AGCAAAAAAGAGGGGCGTCA
promoter	CGACACGATGGCCGCG
narG	CGGCAGGGCTAACCACG
promoter	CCTCCCAGTCCCGGTTTGT
16S	AAATTGAAGAGTTTGATCATGGCTC
rDNA	GCATGGCTGCATCAGGCTT
